# Cerebral microstructural changes in children suffering from hemolytic uremic syndrome

**DOI:** 10.1007/s00431-023-05130-w

**Published:** 2023-08-10

**Authors:** Eva Bültmann, Antonia Zapf, Hans Joachim Mussgnug, Nele Kanzelmeyer, Hans Hartmann

**Affiliations:** 1 Institute of Diagnostic and Interventional Neuroradiology, Hannover Medical School, Carl-Neuberg-Straße 1, D-30625 Hannover, Germany; 2 Institute of Medical Biometry and Epidemiology, University Medical Center Hamburg-Eppendorf, Hamburg, Germany; 3 Clinic for Pediatric Kidney, Liver, and Metabolic Diseases, Hannover Medical School, Hannover, Germany

**Keywords:** Hemolytic uremic syndrome, Magnetic Resonance Imaging, Quantitative Imaging, Diffusion-weighted Imaging

## Abstract

To evaluate microstructural cerebral changes in children suffering from typical hemolytic uremic syndrome (HUS) based on apparent diffusion coefficient (ADC) maps. For 12 pediatric HUS patients (0.8 - 14.6 years of age) conventional magnetic resonance imaging (cMRI) at 1.5 T was retrospectively analyzed. ADC values were measured in 35 different brain regions and compared with age-related, previously published ADC reference values from a healthy pediatric control group. The HUS cohort was divided into 2 subgroups depending on clinical outcome. Subgroup A showed poor neurological outcome whereas subgroup B demonstrated improvement without lasting neurological deficits. Qualitative analysis revealed lesions by diffusion-weighted imaging (DWI) with hypointense correlate on the ADC map in basal ganglia and/or thalami and corresponding T2 hyperintensities in the majority of patients in Subgroup A (80%). Those in Subgroup B did not show qualitative DWI alterations with ADC correlate even when T2 hyperintense lesions were detected in basal ganglia and/or thalami. Quantitative analysis demonstrated abnormal ADC values in all HUS patients with a trend to a greater number of affected regions in Subgroup A compared to Subgroup B (16 versus 11 median number of regions respectively, p = 0.56).

*Conclusion*: Using DWI qualitative and quantitative differences were found between HUS patients showing poor neurological outcome and those without neurological deficits at discharge. While ADC values indicated more extensive cerebral changes than conventional qualitative findings, both may provide early prognostic indicators for neurological outcome in pediatric HUS patients.

**Table:** 

**What is Known:** *• In patients with STEC-HUS and neurological symptoms, MRI may show hyperintense signals on T2 and altered diffusivity mostly affecting basal ganglia, thalami and periventricular white matter.*	
**What is New:** *• In such patients, early MRI including quantitative ADC measurements over different brain regions may allow for detection of signal alterations possibly reflecting microstructural changes in such patients.*

**What is Known:**

*• In patients with STEC-HUS and neurological symptoms, MRI may show hyperintense signals on T2 and altered diffusivity mostly affecting basal ganglia, thalami and periventricular white matter.*

**What is New:**

*• In such patients, early MRI including quantitative ADC measurements over different brain regions may allow for detection of signal alterations possibly reflecting microstructural changes in such patients.*

## Introduction

Typical hemolytic uremic syndrome (HUS) is characterized by the triad of acute renal failure, thrombocytopenia and hemolytic anemia and is mostly caused by Shiga toxin-producing Escherichia coli (STEC) in Europe. Neurological signs are reported in 17 – 52% of cases [1], with Central Nervous System (CNS) involvement contributing significantly to chronic morbidity [2], and potentially to fatal outcome [3]. Magnetic resonance imaging (MRI) may reveal abnormalities affecting the basal ganglia and / or thalami, as well as the deep white matter [4]. Clinical courses of HUS patients with neurological involvement are highly variable, and few prognostic factors have been described to date [3]. Early MRI may offer such options, as postulated by Donnerstag et al. [4].

While renal failure is caused by thrombotic microangiopathy leading to vessel occlusions by platelet–fibrin thrombi [5, 6], the cause of neurological complications is still poorly understood. Several pathophysiological mechanism have been discussed in neurological involvement, such as endothelial injury, thrombus formation, hemorrhage, and posterior reversible encephalopathy syndrome [7, 8]. As Weissenborn et al. [9] and Donnerstag et al. [4] hypothesized, both metabolic and toxic effects may be responsible for CNS involvement. Additionally, Weissenborn et al. [9] indicated microstructural rather than macrostructural cerebral changes, reporting increased T2 relaxation times in basal ganglia in all adult STEC-positive HUS (STEC-HUS) patients showing inconspicuous conventional magnetic resonance imaging (cMRI) on qualitative analysis.

We aimed to define prognostic factors at early stages of the disease, further investigating possible microstructural cerebral alterations in pathological as well as normal appearing brain regions. We retrospectively determined apparent diffusion coefficient (ADC) values in different supra- and infratentorial locations early in the acute phase of neurological involvement in children with STEC-HUS and compared them with age-related ADC reference values from published pediatric controls [10].

## Subjects

This retrospective study was approved by the local institutional review board. Data and neurological symptoms from 12 children suffering from STEC-HUS between 2009 and 2021 and had an MRI at 1.5 T were retrospectively analyzed (5 females, 7 males, median age 2.7 years [range: 0.8–14.6 years]). No patient was excluded. Some patients were previously included in the cohort described by Donnerstag et al. [4] and Pape et al. [11]. Cerebral MRI was performed within 24 h of presentation of neurological symptoms in 6/12 STEC-HUS patients, and between 24 and 48 h in 5/12 STEC-HUS patients to assess severity of structural cerebral alterations and rule out infarction during acute illness. For one STEC-HUS patient (ID 9), MRI was measured 4 days after onset of neurological symptoms. Additionally, four children had an early follow-up MRI within one week and three children received long-term follow-ups between 6 and 18 months. Early follow-up MRI were included only in qualitative analysis. Patients’ clinical courses were variable and we therefore divided our cohort into 2 subgroups depending on outcome. Subgroup A (Patient ID 1, 3, 5, 7 and 8) showed poor neurological outcome with severe neurological deficits including two fatal outcomes, whereas children in subgroup B (Patient ID 2, 4, 6, 9, 10, 11 and 12) clearly improved and were without neurological deficit at discharge. For age-specific comparison with our control subjects [10], children were divided into age-dependent subgroups (0–0.25, 0.25–0.5, 0.5–1, 1–2, 2–3, 3–5, 5–7.5, 7.5–10, 10–12.5 and 12.5–17.2 years); there were no STEC-HUS children in the two youngest age groups and the 7.5–10 years subgroup. The control group comprised 112 children (0–17.2 years) from our pool of pediatric MRI examinations at 1.5 T which were retrospectively selected showing no signal abnormalities [10] Brain MRI was performed for various clinical indications including headache, dizziness and vomiting, sensorineural hearing loss, first epileptic seizures, and newly diagnosed leukemia before therapy. Between 0 and 2 years of age, the age structure of girls and boys was similar without gender-dependent differences in ADC values in any region. In the older age group the age distribution was skewed, so that a gender comparison could not be performed.

## Methods

Routine MRI was performed on 1.5 T MR systems (Avanto and Aera MR systems, Siemens AG or Genesis Signa, GE Medical Systems) and reviewed qualitatively by one experienced neuroradiologist (E.B., more than 15 years of experience). All examinations included axial diffusion-weighted, single-shot, spin-echo echo-planar sequences with 4 mm or 5 mm slices and an imaging matrix = 192 × 192 or 128 × 128) and all but one patient received a SWI or T2* sequence. In three (x, y, z) orthogonal axes an effective b value of 1000 s/mm^2^ was used and additional measurement without diffusion weighting (b = 0 s/mm^2^) was acquired. ADC maps were generated from the scanner. Diffusion-weighted imaging (DWI) and ADC data were transferred to an external workstation for further analysis.

Using manually delineated circular or rectangular regions of interest (ROI) with minimum area 20 mm^2^ (20–283 mm^2^) depending on region and patient anatomy, ADC values were measured in 35 infra- and supratentorial locations as published by Bültmann et al. [10].

ROI position was carefully chosen for anatomically comparable positions on B0 images by the experienced neuroradiologist (E.B.) and transferred to ADC images using the program imageJ (based on Java, public domain https://imagej.nih.gov/ij/). For each location, mean ADC value, standard deviation, as well as minimal and maximal ADC values, were calculated (×10–3 mm^2^/ sec). Two measurements were excluded due to partial volume effects. One patient in subgroup A showed a few small punctate lesion on T2* and one patient developed multiple punctate SWI lesions in the putamen and thalamus on follow-up. These changes were not relevant to the ADC measurements. Control ADC data was also measured at 1.5 T and previously published [10].

## Statistical analysis

Statistical analyses were performed with SAS 9.4 (SAS Institute Inc., Cary, NC, USA). Initially, the control and STEC-HUS groups were compared descriptively relative to age (median and quartiles) and gender (absolute and relative frequencies). The STEC-HUS cohort was then described individually and in subgroups depending on clinical outcome (median age and number of involved regions with comparison by exact Wilcoxon two-sample test). To compare age-dependent ADC values for STEC-HUS and control children, values for the former were evaluated individually as below, within or above the reference range (taken from [10]), and corresponding absolute and relative frequencies were calculated.

## Results

**Fig. 1:**
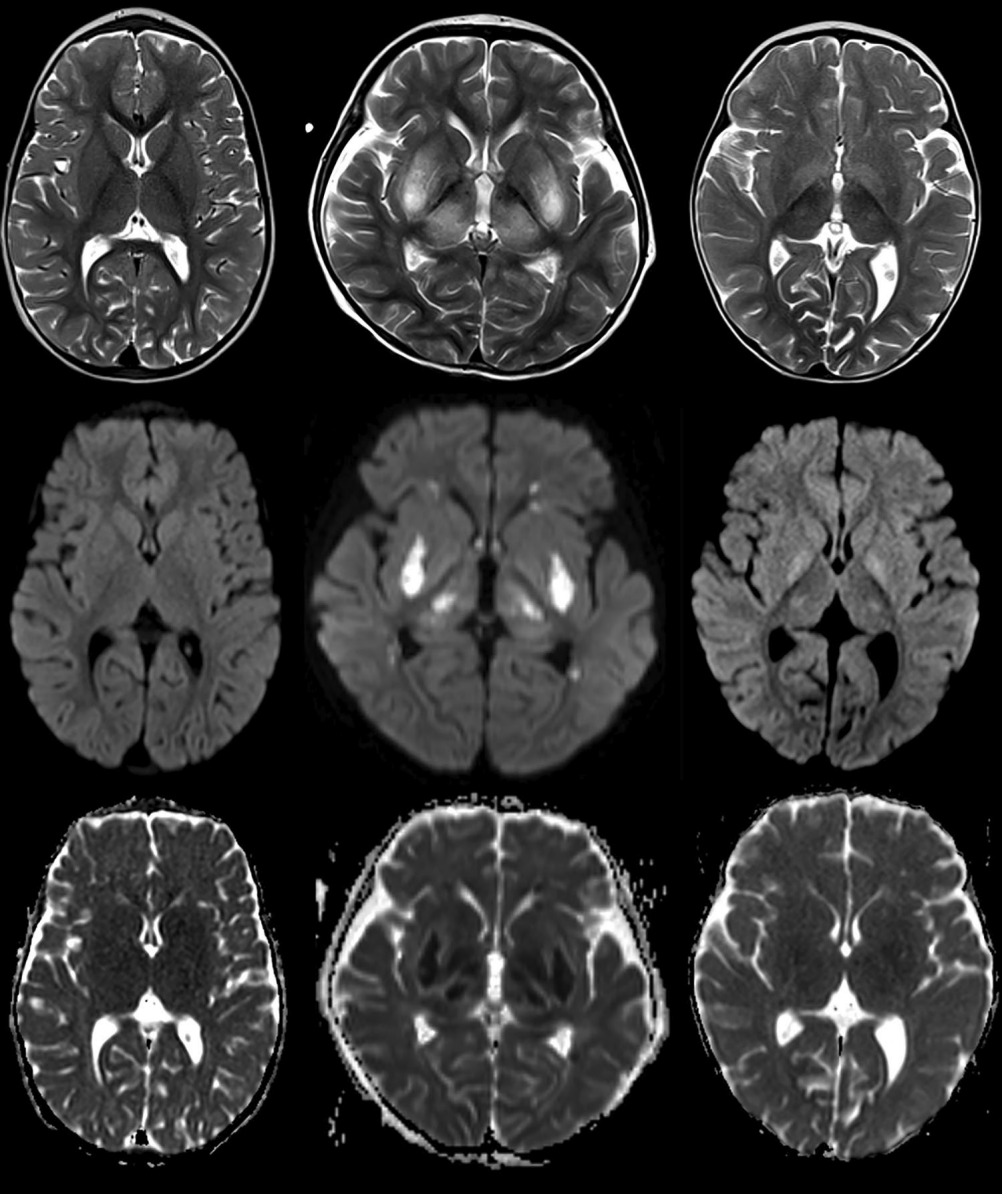
Qualitative review of cMRI scans showed different pattern: Normal T2w, DWI and ADC images in the control group (left row), bilateral T2 hyperintensities in basal ganglia and thalami with corresponding reduced diffusivity in subgroup A (middle row) and bilateral T2 hyperintensities with T2 shine through on DWI in subgroup B without corresponding signal abnormalities on ADC (right row)
- Quantitative comparison of ADC values for STEC-HUS and control subjects and between STEC-HUS subgroups
  Overall, in 154 measurements (38%) in STEC-HUS patients mean ADC values fell outside the age-dependent reference range [10] (Table 2). For each STEC-HUS patient, this occurred in at least 4 regions. In total, mean ADC was decreased in 112 measurements (27%) and increased in 42 measurements (10%) for STEC-HUS patients compared to the control group (Table 3). With respect to number of regions showing altered ADC values, we observed a trend towards more regions being involved in patients in Subgroup A with poor neurological outcome in comparison to Subgroup B with favorable neurological outcome (16 vs 11 median number of regions, p = 0.56).

- Demographic and baseline characteristics
  Gender and median age were similarly distributed for STEC-HUS patients (n=12, 5 female, 42%; median age 2.7 years, age range 0.8-14.6 years) and healthy control subjects (n=112, 52 female, 46%; median age 3.3 years, age range 0-17.2 years). With regard to age, both subgroups were comparable (median age 2.3 years for subgroup A compared to 3 years for subgroup B, p = 0.56).
- Qualitative review of cMRI scans
  In 8/12 STEC-HUS children (including Patient 8 on follow-up MRI 4 days after onset), T2 hyperintense lesions in basal ganglia and/ or thalami were found, including all 5 patients in Subgroup A with poor neurological outcome (Table 1). Except for Patient 3, all patients in Subgroup A showed corresponding lesions on DWI with hypointense ADC correlate which means reduced diffusivity in basal ganglia and /or thalami (Patient 8 on early follow-up MRI). Children in Subgroup B without neurological sequelae did not show qualitative DWI alterations with corresponding ADC hypointensities in these regions. For one patient (Patient 12), there was T2 hyperintensity, but no DWI or ADC changes. For two (Patients 9 and 10), there were T2 hyperintense alterations within the basal ganglia and corresponding DWI hyperintensities, but no signal reductions on ADC which correspond to T2 shine through. Further, Patient 10 had punctate lesions with reduced diffusivity in the supratentorial white matter on the first MRI scan, which largely resolved on early follow-up. Figure 1 compares the different pattern.

**Table 1:** Qualitative imaging findings in basal ganglia/ thalami for STEC-HUS children. T2 hyperintense basal ganglia/thalamic lesions with corresponding reduced diffusivity were only found in subgroup A with poor neurological outcome

**Patient ID**	**Age (years)**	**Subgroup**	**Basal ganglia and/or thalamic alterations**	
			**Hyperintense T2 lesions**	**DWI lesions**
1	1.4	A	+	+ (DWI ⇧, ADC ⇩)
1 FU	1.4	A	+	+ (DWI ⇧, ADC ⇩)
3	2.3	A	+	- (DWI ⇧, ADC =)
5	7.3	A	+	+ (DWI ⇧, ADC ⇩)
7	3	A	+	+ (DWI ⇧, ADC ⇩)
8	0.8	A	-	-
8 FU	0.8	A	+	+ (DWI ⇧, ADC ⇩)
2	1.8	B	-	-
4	10	B	-	-
6	14.6	B	-	-
9	1.2	B	+	- (DWI ⇧, ADC =)
10	3	B	+	- (DWI ⇧, ADC =)
10 FU	3	B	-	-
11	1.2	B	-	-
12	3.5	B	+	-
12 FU	3.5	B	+	-

DWI ⇧, DWI ⇩, DWI = : hyperintense, hypointense or isointense signal on diffusion-weighted images

ADC ⇧, ADC ⇩, ADC = : hyperintense, hypointense or isointense signal on ADC map

FU early Follow-up

**Table 2:** 154 quantitative ADC measurements (×10–3 mm2/s) which fell outside the age-dependent reference range (left columns) and the mean ADC value as well as the reference range of the control group (right 3 columns)

**ID**	**Age**	**Sex**	**Subgroup**	**Region**	**Mean ADC value**		**Mean ADC value**	**Lower reference limit**	**Upper reference limit**
1	1,4	mM	A	1	**0,545359**		0,932230714	0,857574026	1,006887403
1	1,4	m	A	2	**0,623391**		0,93774	0,879029704	0,996450296
1	1,4	m	A	6	**0,7615**		0,831696857	0,77410735	0,889286365
1	1,4	m	A	7	**0,744248**		0,805954	0,752799737	0,859108263
1	1,4	m	A	9	**0,6475**		0,794077	0,728225332	0,859928668
1	1,4	m	A	10	**0,708917**		0,794042286	0,738872735	0,849211836
1	1,4	m	A	13	**0,55688**		0,904187357	0,842305559	0,966069155
1	1,4	m	A	14	**0,65692**		0,898273214	0,826983812	0,969562617
1	1,4	m	A	15	**0,824031**		0,91016857	0,864432111	0,959601603
1	1,4	m	A	20	**0,494705**		0,813899857	0,714122673	0,913677042
1	1,4	m	A	21	**0,55141**		0,908115714	0,758309461	1,057921968
1	1,4	m	A	22	**0,566908**		0,854883286	0,642312318	1,067454253
1	1,4	m	A	23	**0,578335**		0,784978357	0,640369928	0,929586786
1	1,4	m	A	24	**0,630898**		0,786086571	0,64032465	0,931848493
1	1,4	m	A	25	**0,48092**		0,791603357	0,691444017	0,891762698
1	1,4	m	A	26	**0,501009**		0,802232929	0,690398128	0,914067729
1	1,4	m	A	29	**0,697029**		0,903935214	0,831920489	0,97594994
1	1,4	m	A	30	**0,735609**		0,868666214	0,824061874	0,913270555
1	1,4	m	A	31	**0,703846**		0,873125143	0,83250633	0,913743956
1	1,4	m	A	32	**0,73187**		0,891506929	0,801030126	0,981983731
1	1,4	m	A	33	**0,780449**		0,879554143	0,810472038	0,948636247
2	1,8	m	B	1	**0,845333**		0,932230714	0,857574026	1,006887403
2	1,8	m	B	2	**0,78521**		0,93774	0,879029704	0,996450296
2	1,8	m	B	6	0,900275		0,831696857	0,77410735	0,889286365
2	1,8	m	B	8	0,861786		0,805321143	0,756213107	0,854429179
2	1,8	m	B	13	**0,83375**		0,904187357	0,842305559	0,966069155
2	1,8	m	B	14	**0,803859**		0,898273214	0,826983812	0,969562617
2	1,8	m	B	15	**0,84505**		0,912016857	0,864432111	0,959601603
2	1,8	m	B	19	1,036806		0,846868643	0,696846501	0,996890784
2	1,8	m	B	21	**0,744033**		0,908115714	0,758309461	1,057921968
2	1,8	m	B	29	**0,72575**		0,903935214	0,831920489	0,97594994
2	1,8	m	B	30	**0,756119**		0,868666214	0,824061874	0,913270555
2	1,8	m	B	32	1,057178		0,891506929	0,801030126	0,981983731
3	2,3	m	A	1	**0,829588**		0,921773462	0,850082487	0,993464436
3	2,3	m	A	2	**0,834625**		0,923531462	0,850251366	0,996811557
3	2,3	m	A	15	**0,829475**		0,907640769	0,83458854	0,980692999
3	2,3	m	A	16	**0,7682**		0,903763308	0,84216951	0,965357106
3	2,3	m	A	29	**0,697267**		0,869158846	0,823057807	0,915259885
3	2,3	m	A	30	**0,746933**		0,850617538	0,768189556	0,933045521
3	2,3	m	A	31	**0,720849**		0,849866385	0,757333606	0,942399164
3	2,3	m	A	32	**0,744735**		0,856067077	0,776509369	0,935624785
4	10	f	B	1	0,897		0,807277889	0,735512874	0,879042904
4	10	f	B	5	**0,688294**		0,728497333	0,709513738	0,747480929
4	10	f	B	16	**0,764562**		0,844174111	0,766689994	0,921658229
4	10	f	B	17	0,932375		0,852164667	0,781045546	0,923283787
4	10	f	B	18	0,9455		0,853638222	0,777056552	0,930219892
4	10	f	B	19	**0,700125**		0,8183785	0,709626614	0,927130386
4	10	f	B	20	**0,648625**		0,81846725	0,77155634	0,86537816
4	10	f	B	26	0,784833		0,714421222	0,660456235	0,768386209
4	10	f	B	28	0,838667		0,755458667	0,717387875	0,793529458
4	10	f	B	35	0,906562		0,815549222	0,766773287	0,864325157
5	73	m	A	2	**0,74282**		0,8320362	0,750682302	0,913390098
5	73	m	A	7	**0,638321**		0,7434887	0,715198749	0,771778651
5	73	m	A	8	**0,636**		0,7418824	0,693775059	0,789989741
5	73	m	A	9	**0,664868**		0,7680451	0,705028167	0,831062033
5	73	m	A	10	**0,67114**		0,7520969	0,688786512	0,815407288
5	73	m	A	11	**0,718**		0,8004056	0,736947371	0,863863829
5	73	m	A	13	**0,709482**		0,8611605	0,812584799	0,909736201
5	73	m	A	14	**0,720343**		0,8540004	0,812437824	0,895562976
5	73	m	A	15	0,993017		0,8637822	0,794729818	0,932834582
5	73	m	A	16	1,021333		0,8662852	0,807177023	0,925393377
5	73	m	A	17	0,956795		0,8898824	0,826130122	0,953634678
5	73	m	A	21	**0,716341**		0,823287625	0,742451781	0,904123469
5	73	m	A	26	**0,670517**		0,765817667	0,696748422	0,834886912
5	73	m	A	27	0,889764		0,7403509	0,67175798	0,80894382
5	73	m	A	28	**0,631705**		0,7839893	0,719484643	0,848493957
5	73	m	A	29	**0,686908**		0,797439222	0,731767539	0,863110905
5	73	m	A	30	**0,704431**		0,7634778	0,710485161	0,816470439
5	73	m	A	32	**0,683247**		0,8153246	0,704226853	0,926422347
5	73	m	A	33	**0,684579**		0,8212054	0,6973857	0,9450251
5	73	m	A	35	0,87682		0,8368625	0,805455582	0,868269418
6	14,6	f	B	24	0,83045		0,697248882	0,643410667	0,751087098
6	14,6	f	B	27	0,838667		0,764548588	0,697828928	0,831268248
6	14,6	f	B	34	**0,754357**		0,813047353	0,772601934	0,853492772
6	14,6	f	B	35	**0,725643**		0,810225059	0,765998543	0,854451575
7	3	f	A	3	1,077773		0,8045955	0,764793912	0,844397088
7	3	f	A	4	0,941568		0,7998456	0,75423152	0,84545968
7	3	f	A	5	1,239517		0,7863556	0,737112384	0,835598816
7	3	f	A	6	1,298841		0,7852403	0,727433952	0,843046648
7	3	f	A	8	0,951404		0,7884884	0,743014538	0,833962262
7	3	f	A	9	1,105953		0,7798529	0,719864027	0,839841773
7	3	f	A	10	1,123506		0,7728439	0,705704407	0,839983393
7	3	f	A	11	1,194977		0,8166827	0,748422232	0,884943168
7	3	f	A	12	1,321827		0,824824	0,748388425	0,901259575
7	3	f	A	18	0,983		0,9025253	0,830688026	0,974362574
7	3	f	A	24	0,84602		0,7333032	0,64118609	0,82542031
7	3	f	A	28	1,17724		0,8333407	0,740876665	0,925804735
7	3	f	A	29	**0,69386**		0,8405815	0,741372665	0,939790335
7	3	f	A	33	0,987127		0,8341331	0,749985007	0,918281193
7	3	f	A	34	1,084845		0,8431069	0,790803276	0,895410524
7	3	f	A	35	1,054052		0,8484378	0,764414689	0,932460911
8	0,8	f	A	9	**0,734353**		0,839462727	0,738973396	0,939952058
8	0,8	f	A	10	**0,687048**		0,837704818	0,729227191	0,946182445
8	0,8	f	A	17	1,069864		0,962256636	0,884493646	1,040019627
8	0,8	f	A	28	**0,841033**		1,026523182	0,895712074	1,15733429
8	0,8	f	A	29	**0,887851**		1,024459364	0,912073989	1,136844738
8	0,8	f	A	32	**0,760552**		0,995957182	0,883247806	1,108666558
8	0,8	f	A	33	**0,854144**		1,001011727	0,876255288	1,125768167
9	1,2	f	B	3	**0,774762**		0,854555643	0,776055895	0,933055391
9	1,2	f	B	4	**0,76099**		0,851428643	0,783701814	0,919155472
9	1,2	f	B	6	**0,729907**		0,831696857	0,77410735	0,889286365
9	1,2	f	B	7	**0,70104**		0,805954	0,752799737	0,859108263
9	1,2	f	B	8	**0,709043**		0,805321143	0,756213107	0,854429179
9	1,2	f	B	9	**0,701908**		0,794077	0,728225332	0,859928668
9	1,2	f	B	10	**0,706597**		0,794042286	0,738872735	0,849211836
9	1,2	f	B	11	**0,751127**		0,832493643	0,770737824	0,894249461
9	1,2	f	B	12	**0,730756**		0,832044	0,768263519	0,895824481
9	1,2	f	B	15	**0,820855**		0,912016857	0,864432111	0,959601603
9	1,2	f	B	16	**0,816974**		0,9127945	0,845429143	0,980159857
9	1,2	f	B	28	**0,789136**		0,907861571	0,835494678	0,980228465
9	1,2	f	B	30	**0,817808**		0,868666214	0,824061874	0,913270555
9	1,2	f	B	31	**0,773587**		0,873125143	0,83250633	0,913743956
10	3	m	B	1	**0,680883**		0,8931099	0,806565474	0,979654326
10	3	m	B	2	**0,680305**		0,8799149	0,80148193	0,95834787
10	3	m	B	3	**0,726139**		0,8045955	0,764793912	0,844397088
10	3	m	B	4	**0,710623**		0,7998456	0,75423152	0,84545968
10	3	m	B	5	**0,641382**		0,7863556	0,737112384	0,835598816
10	3	m	B	6	**0,637612**		0,7852403	0,727433952	0,843046648
10	3	m	B	7	**0,562698**		0,7835654	0,736865877	0,830264923
10	3	m	B	8	**0,582853**		0,7884884	0,743014538	0,833962262
10	3	m	B	9	**0,640886**		0,7798529	0,719864027	0,839841773
10	3	m	B	10	**0,67047**		0,7728439	0,705704407	0,839983393
10	3	m	B	11	**0,704901**		0,8166827	0,748422232	0,884943168
10	3	m	B	12	**0,708983**		0,824824	0,748388425	0,901259575
10	3	m	B	13	**0,586011**		0,8659601	0,821579074	0,910341126
10	3	m	B	14	**0,577367**		0,8477702	0,804048171	0,891492229
10	3	m	B	16	**0,754663**		0,8891638	0,7939837	0,9843439
10	3	m	B	18	0,998983		0,9025253	0,830688026	0,974362574
10	3	m	B	20	**0,543167**		0,79533225	0,730493503	0,860170997
10	3	m	B	25	**0,642965**		0,7607909	0,694651881	0,826929919
10	3	m	B	28	**0,548985**		0,8333407	0,740876665	0,925804735
10	3	m	B	29	**0,535269**		0,8405815	0,741372665	0,939790335
10	3	m	B	30	**0,465462**		0,8250045	0,739148099	0,910860901
10	3	m	B	31	**0,449267**		0,8228115	0,745148477	0,900474523
10	3	m	B	32	**0,511009**		0,8400721	0,750709979	0,929434221
10	3	m	B	33	**0,528109**		0,8341331	0,749985007	0,918281193
11	1,2	m	B	1	**0,843359**		0,932230714	0,857574026	1,006887403
11	1,2	m	B	2	**0,876014**		0,93774	0,879029704	0,996450296
11	1,2	m	B	28	**0,816626**		0,907861571	0,835494678	0,980228465
11	1,2	m	B	30	**0,820661**		0,868666214	0,824061874	0,913270555
11	1,2	m	B	31	**0,757744**		0,873125143	0,83250633	0,913743956
11	1,2	m	B	32	**0,760667**		0,891506929	0,801030126	0,981983731
11	1,2	m	B	33	**0,755933**		0,879554143	0,810472038	0,948636247
12	35	m	B	3	0,890824		0,8045955	0,764793912	0,844397088
12	35	m	B	8	**0,739677**		0,7884884	0,743014538	0,833962262
12	35	m	B	1,8	0,97925		0,9025253	0,830688026	0,974362574
12	35	m	B	20	0,907983		0,79533225	0,730493503	0,860170997
12	35	m	B	24	0,830519		0,7333032	0,64118609	0,82542031
12	35	m	B	25	0,841444		0,7607909	0,694651881	0,826929919
12	35	m	B	26	0,835069		0,7517141	0,697707652	0,805720548
12	35	m	B	29	**0,708343**		0,8405815	0,741372665	0,939790335
12	35	m	B	30	**0,727263**		0,8250045	0,739148099	0,910860901
12	35	m	B	34	0,909828		0,8431069	0,790803276	0,895410524
12	35	m	B	35	0,934741		0,8484378	0,764414689	0,932460911

Reduced ADC values are highlighted in bold type

**Table 3:** Frequencies of ADC values below and above the age-dependent reference range for each STEC-HUS subgroup and median number of involved regions (with 25% and 75% quantile)

**Subgroup**	**Number of regions with altered ADC values**			**Median number of involved regions**
	**Below**	**Above**	**Total**	
**A**	51 (29%)	21 (12%)	72 (41%)	16 (8; 20)
**B**	61 (25%)	21 (9%)	82 (34%)	11 (7; 14)
**Total A + B**	112 (27%)	42 (10%)	154 (38%)

## Discussion

Although CNS involvement frequently complicates typical STEC-HUS, microangiopathy and small vessel involvement have rarely been clearly demonstrated in patients [12–14]. Our group [4, 9] has previously discussed possible metabolic and / or toxic contribution and imaging abnormalities on cMRI scans in STEC-HUS patients. In addition to macrostructural changes, Weissenborn et al. [9] described microstructural alterations. For all adult STEC-HUS patients with normal cMRI scans using quantitative MRI parameters, significantly elongated T2 relaxation times in basal ganglia were reported compared to healthy controls but ADC values were unchanged. In contrast, Donnerstag et al. [4] measured ADC values in infra- and supratentorial lesions on diffusion-weighted images in STEC-HUS children. They found increased as well as decreased intralesional ADC values compared to age-related values obtained from Morriss et al. [15]. Thus, the question arose as to whether we could detect ADC value alterations in STEC-HUS children as an indicator of microstructural changes independent of findings on cMRI scans.

In the present study, we present data for a cohort of 12 children with typical STEC-HUS and clinical CNS involvement divided into two subgroups depending on neurological outcome. Qualitatively, the majority of our STEC-HUS patients (67%) showed T2 hyperintense lesions in basal ganglia and/ or thalami. The presence of T2 hyperintense lesions together with corresponding reduced diffusivity in these brain regions was only observed in patients with poor outcome. Quantitative assessment of ADC values in multiple standardized brain regions showed ADC abnormalities involving brain structures that appeared normal on qualitative evaluation. These findings were most pronounced in patients with devastating neurological outcome.

In accordance with Donnerstag et al. [4], but in contrast to Gitiaux et al. [16], we also measured decreased ADC values, as a sign of limited diffusion in cytotoxic edema, as well as increased ADC values, as part of a vasogenic edema. However, Gitiaux et al. [16] performed intra-individual comparisons of ADC values between involved and normal appearing areas, while we compared values for STEC-HUS children with those from a pediatric control group of children with age-appropriate development.

Using ADC values, we could identify more extensive changes in comparison to qualitative lesions detected by cMRI. ADC alterations were measured in normal appearing brain regions on T2w-, T1w- and FLAIR images indicating a more widespread involvement of the brain. Although Weissenborn et al. [9] did not report such changes, it should be noted that their study involved adults, while we investigated children. As described in several publications [17–19], development of the human brain extends through to adulthood, with myelination an essential phase of postnatal brain maturation [20]. In accordance, ADC and T2 values decrease until late childhood [9, 21, 22]. Some publications have already considered whether increasing T2 hypointensity during brain maturation may reflect chemical maturation of the myelin sheath, involving decreasing water content, increasing hydrophobicity [23] and tightening of the myelin spiral around the axon [24]. These ongoing maturational processes could underly a higher vulnerability in the pediatric brain and contribute to differences between child and adult STEC-HUS patients.

When considering neurological outcome, basal ganglia and thalamic lesions on diffusion-weighted images appeared to be a prognostic factor for poor outcome in our children, in particular in association with corresponding T2 hyperintensities in the same location. Gitiaux et al. [16] recorded the most severe T2 hyperintensities and the highest ADC alterations in basal ganglia of two patients with a fatal outcome. Weissenborn et al. [9] also measured quantitative basal ganglia involvement in terms of T2 relaxation times in patients suffering from STEC-HUS neurological complications. Taken together, these studies along with our present data indicate a significant role for basal ganglia involvement in STEC-HUS. In a rat model, Goldstein et al. [25] demonstrated direct effects of Shiga toxin 2 in neurons and glial cells after intracerebroventricular microinfusion and confirmed Shiga toxin 2 presence in different striatum cell compartments, supporting our hypothesis. On the contrary, Nathanson et al. [26] reported that neurological outcome did not depend on localized cerebral involvement. However, the latter neither described how CNS damage on MRI was defined or included quantitative imaging. MRI was mostly obtained at a later timepoint after disease onset, which may influence signal changes. Early MRI was performed in 22/29 patients later than 2 days after onset of neurological symptoms, in contrast to 1/12 in our cohort. A recent retrospective study of pediatric patients with STEC-HUS including 22 with neurological involvement showed good neurological outcome in spite of DWI abnormalities involving the deep white matter and grey matter. Similarly to our observation, the authors also described DWI abnormalities with mixed increased and decreased ADC values [27], and good outcome in most patients in spite of DWI abnormalities, including 2/3 with involvement of basal ganglia. However, the study does not include information on timing of investigation and most importantly quantitative ADC values. Therefore, the results cannot be directly compared to our findings and do not contradict our observation that basal ganglia and thalamic lesions on diffusion-weighted images appeared to be a prognostic factor for poor outcome.

Our present study further indicates that poorer prognosis is associated with involvement of a greater number of regions showing quantitative ADC value alterations reflecting a more widespread, extensive brain involvement. No relevant differences were seen in the ratio of ADC values below versus above the normal range (Subgroup A 2.4: 1; Subgroup B 2.9:1). Gitiaux et al. [16] described milder and less widespread hyperintensities on T2/FLAIR images compared to DWI abnormalities. We suggest quantitative assessment of diffusion-weighted images may predict neurological outcome in acute disease and would therefore be required as soon as practicable after presentation of neurological symptoms.

While age did not significantly differ between STEC-HUS subgroups, children with a poor prognosis tended to be younger, in accordance with previous literature [28]. Age at STEC-HUS onset may be relevant, possibly linked to a greater sensitivity of the more immature brain.

Our study has practical limitations. While the number of STEC-HUS children is low, as typical HUS is rare and neurological involvement is only observed in a third of cases [3], achieving a large series would be highly challenging. Consequently, with a small cohort and age dependency of ADC values, we were confined to descriptive analysis. We also compared patient ADC values with published control reference values [10], which, though not verified from additional studies, are comparable with data from the literature [29–31].

For the first time using early MRI and quantitative ADC analysis, we were able to demonstrate changes in otherwise normal appearing brain regions in STEC-HUS children with neurological involvement. We were also able to define potential predictive factors that require ratification in further imaging studies including different quantitative parameters. Predictors of poor prognosis are necessary to guide treatments such as the C5-complement inhibitor Eculizumab [11].

In conclusion, our results suggest that DWI and quantitative ADC value measurements are able to detect subtle microstructural changes in STEC-HUS children, which are in part not visible on cMRI. This may add to the understanding of pathophysiological mechanisms involved in neurological involvement. Involvement of deep gray matter structures on DWI and T2w images may provide key prognostic factors, though this needs to be confirmed in larger case series.

## Data Availability

The data are available on request.
